# Plain Language Summary on The European Union One Health 2022 Zoonoses Report

**DOI:** 10.2903/j.efsa.2023.p211202

**Published:** 2023-12-12

## Abstract

This publication is linked to the following EFSA Journal article: https://efsa.onlinelibrary.wiley.com/doi/10.2903/j.efsa.2023.8442
